# Incidence and risk of hematologic toxicities in cancer patients treated with regorafenib

**DOI:** 10.18632/oncotarget.21217

**Published:** 2017-09-23

**Authors:** Bin Zhao, Hong Zhao

**Affiliations:** ^1^ Center for Scientific Research, The Second Affiliated Hospital & Yuying Children’s Hospital, Wenzhou Medical University, Wenzhou, 325027, China; ^2^ Department of Medical Oncology, The Third Affiliated Hospital of Harbin Medical University, Harbin, 150081, China

**Keywords:** regorafenib, hematologic toxicity, cancer, adverse event

## Abstract

Regorafenib, an oral vascular endothelial growth factor receptor tyrosine-kinase inhibitor, has been approved for the treatment of several malignancies. As a non-traditional cytotoxic chemotherapeutic agent, regorafenib is often associated with hematologic toxicities. Here we searched PubMed and Embase up to June 2017 for relevant clinical trials. Eligible studies include trials in which subjects treated with 160 mg of regorafenib daily during the first 21 days of each 28-day cycle, and adequate safety data profile reporting thrombocytopenia, anemia, neutropenia and leucopenia. Statistical analyses were conducted to calculate the overall incidences, relative risks (RRs) and their 95% confidence intervals (CIs). A total of 2,341 subjects from 16 trials were included in the present studies. The incidences of regorafenib associated all-grade and high-grade hematologic toxicities were: thrombocytopenia, 22% and 3%; anemia, 20% and 3%; neutropenia, 10% and 2%, and leucopenia, 13% and 2%, respectively. Regorafenib-treated subjects had a significant increased risk of all-grade (RR=6.35; 95% CI, 3.19-12.64) and high-grade (RR=6.27; 95% CI, 1.69-23.26) thrombocytopenia, all-grade (RR=2.76; 95% CI, 1.63-4.68) and high-grade (RR=5.38; 95% CI, 1.60-18.06) anemia. Our results suggested that regorafenib therapy was associated with significantly increased risks of hematological toxicities, and hematologic monitoring at regular intervals should be advised to clinician.

## INTRODUCTION

Tyrosine kinase inhibitors (TKIs) are small molecules that bind to the activation domain of tyrosine kinase receptors, and have emerged as an important kind of anti-cancer agents. Regorafenib (also referred as Stivarga, BAY 73-4506) can inhibit the activity of angiogenic, stromal and oncogenic tyrosine kinases by targeting vascular endothelial growth factor receptors 1, 2, 3 (VEGFR1, VEGFR2 and VEGFR3), tyrosine protein kinase receptor Ret, tyrosine-protein kinase TIE-2, basic fibroblast growth factor receptor-1, platelet-derived growth factor beta, proto-oncogene RAF-1, c-KIT, BRAF and p38 MAP kinase [1, 2]. Currently, it has been approved by the United States Food and Drug Administration (FDA) for the treatment of metastatic colorectal cancer (CRC) [3], advanced gastrointestinal stromal tumor (GIST) [4] and recently, advanced hepatocellular carcinoma (HCC) [5].

Compared with traditional cytotoxic chemotherapeutic agents, VEGF-targeted TKIs, such as regorafenib, sunitinib and sorafenib, are associated with a distinct profile of adverse events. Previous studies have shown an increased risk of developing hypertension [6], hepatic toxicity [7], hand-foot skin reaction [8] and arterial thromboembolism [9] in patients treated with VEGF-TKIs. In addition, the significant risk of hematologic toxicities associated with sunitinib [10], sorafenib [11], bevacizumab [12] and ramucirumab [13] have been systematically analyzed. Pre-clinical studies have revealed that VEGF and its receptor play a key role in hematopoiesis by regulating hematopoietic stem cells cycling, differentiation and hematopoietic recovery [14, 15]. In addition, hematopoietic stem cells express both VEGFR-1 and VEGFR-2 [16, 17], and are essential for the migration of these cells [18].

Currently, regorafenib is being investigated in several types of tumors and an increase in the application of regorafenib could be expected in the near future. However, although hematologic toxicities associated with regorafenib have been reported in numerous studies, there has been no systematic attempt to synthesize these data and the overall risk of hematologic toxicities induced by regorafenib has yet to be assessed. Accordingly, here we conducted a systematic review and meta-analysis of available clinical studies to determine the overall incidence and risk of developing hematologic toxicities in subjects treated with regorafenib.

## RESULTS

### Search results

**Figure 1:**
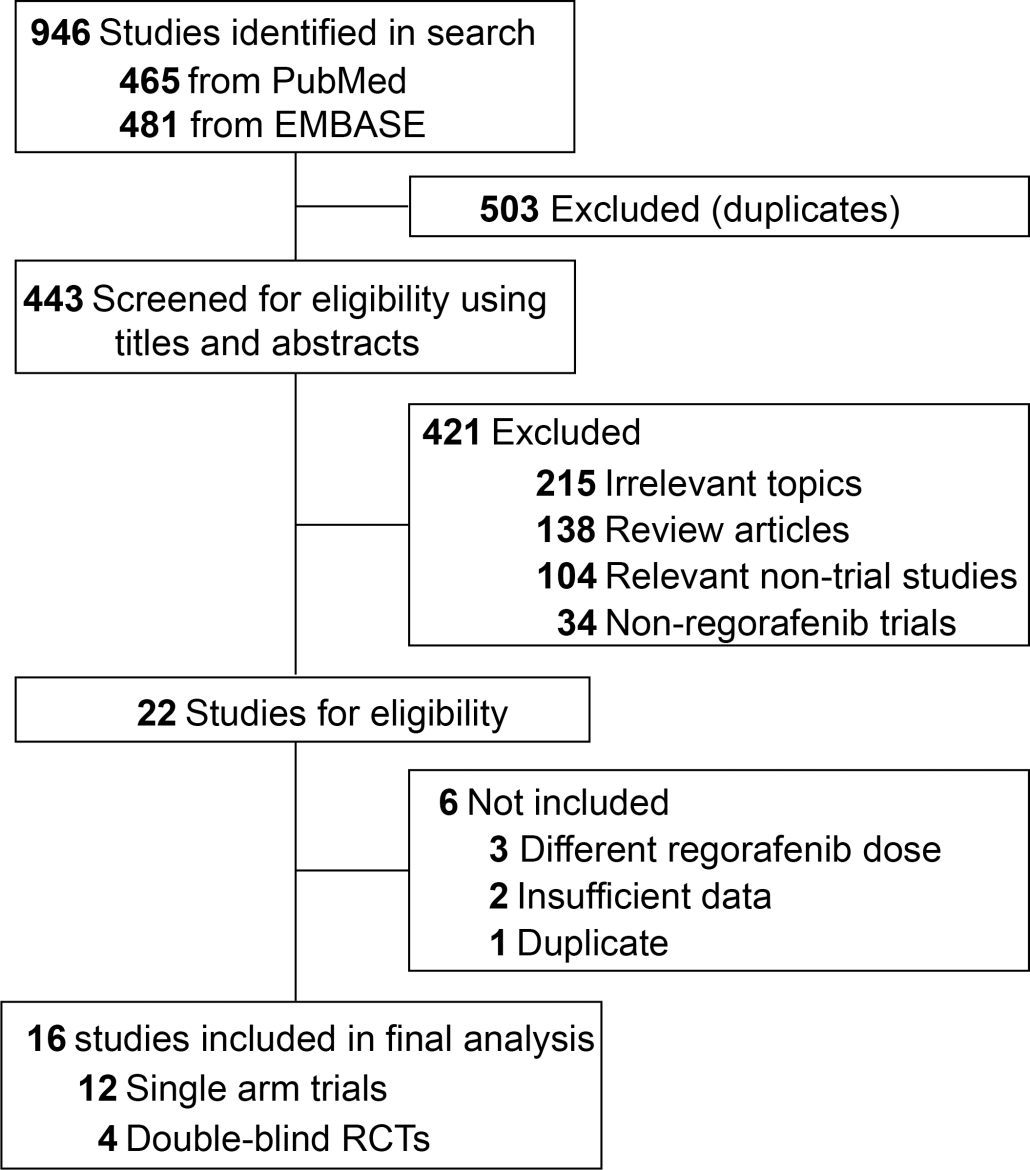
Flow-chart diagram of selected trials included in this meta-analysis

A total of 946 potentially relevant studies were identified by the initial search strategy, including 465 articles on regorafenib from PubMed and 481 papers from EMBASE database. 503 studies were removed because of duplications. By screening of the titles and abstracts, 421 articles were excluded because they did not satisfy the inclusion criteria. After carefully reviewed the full texts of the remaining 22 potentially eligible papers, 6 more were exclude because of insufficient data (n=2) [19, 20], different dose of regorafenib (n=3) [21–23] and duplication (n=1) [24]. A total of 16 studies were selected for the final analysis. 12 studies were single arm trials [25–36], the other 4 were randomized, placebo-controlled trials [37–40]. A flow chart showing the study selection was presented in Figure 1.

### Study quality

All included phase III trials involved randomized treatment allocation [37–39]. Of the rest 13 trials, 12 trials were single-arm trials [25–36], while one was double-blind, randomized trials. For quality analysis purposes, we calculated the incidences in randomized trials versus non-randomized trials and phase III RCTs versus non-phase III studies. We found no statistically significant difference between these subgroup analysis (Data not shown).

### Population characteristics

A total of 2,341 subjects were included in this study (regorafenib: 1,735; control: 606). 1,466 of these patients had CRC (regorafenib: 1,145; control: 321) from 10 trials. 603 patients had HCC (regorafenib: 410; control: 193) from 2 trials. 75 subjects had GIST (regorafenib: 75; control: 0) from 2 trials. 182 patients had soft tissue sarcoma (STS; regorafenib: 90; control: 92) from 1 trial. The schedule and dose of regorafenib for all trials were 160 mg once daily orally for the first 21 days of each 28-day cycle, the current FDA-recommended dose until disease progression or unacceptable toxicity. The median treatment ranged from 1.6 months [38] to 10.0 months [34]. The clinic-pathological characteristics of eligible studies were summarized in Table 1. The numbers of all-grade and high-grade events for each trial were presented in Table 2. It should be noted that not all trials consistently reported the four hematologic adverse events of our interest.

**Table 1:** Baseline characteristics of the clinical trials included in this study

Author	Region	Year	Underlying malignancy	Follow-up, median (range), month	No. of patients	Median age (range), year	Gender (male/female)	ECOG PS (0/1/2)	Treatment duration, median (range), month	Median OS (95% CI), month	Median PFS (95% CI), month
Li [39]	Asia	2015	CRC	7.4(4.3-12.2)	136 68	58(50-66) 56(49-62)	85/51 33/35	35/101/0 15/53/0	2.4(1.6-5.3) 1.6(1.1-1.6)	8.8(7.3-9.8) 6.3(4.8-7.6)	3.2(2.0-3.7) 1.7(1.6-1.8)
Grothey [38]	Globe	2013	CRC	NR	505 255	61(54-67) 61(54-68)	311/194 153/102	265/240/0 146/109/0	1.7(1.4-3.7) 1.6(1.3-1.7)	NR NR	1.9(1.6-3.9) 1.7(1.4-1.9)
Mir [40]	France	2016	STS	16.8(14.4-19.8)^*^	90 92	56(21-81) 54(20-80)	43/47 49/43	41/49/0 45/46/1	3.1(0.6-10.8) 2.1(0.7-5.1)	13.4(8.6-17.3) ^*^ 9.0(6.8-12.5)	4.0(2.6-5.5) ^*^ 1.0(0.9-1.8)
Bruix [37]	Globe	2017	HCC	7.0(3.7-12.6)	379 194	64(54-71) 62(55-68)	333/46 171/23	247/132/0 130/64/0	3.6(1.6-7.6) 1.9(1.4-3.9)	10.6(9.1-12.1) 7.8(6.3-8.8)	3.1(2.8-4.2) 1.5(1.4-1.6)
Argiles [25]	Globe	2015	CRC	NR	53	61(32-80)	28/26	35/19/0	7.7(0.1-19.5)	NR	8.5(7.4-11.3)
Masuishi [31]	Japan	2017	CRC	6.5	146	NR	90/56	135/11^**^	NR	6.7(5.8-7.6)	2.1(1.8-2.5)
Calcagno [27]	France	2016	CRC	NR	29	68(40-83)	NR	7/18/4	2.5(0.1-11.4)	6.0(5.0-8.0)	NR
Del Prete [28]	Italy	2017	CRC	NR	136	57(31-79)	92/44	104/32^**^	3.5	8.9	2.8
Yeh [34]	Taiwan	2017	GIST	4.0	18	59(36-71)	14/4	6/12/0	10.0(0.6-24.9)	10.9(1.0-27.0)	22.1
Kim [29]	Korea	2015	CRC	NR	32	57(29-79)	20/12	31/1^**^	NR	NR	4.2(3.1-5.2)
Son [33]	Korea	2017	GIST	12.7(0.2-27.6)	57	56(50-62)	34/23	0/52/5	4.7(0.9-27.1)	12.9(8.1-17.7)	4.5(3.8-5.3)
Zanwar [35]	India	2016	CRC	NR	23	50	12/11	2/15/6	3.8	NR	NR
Bruix [26]	Globe	2013	HCC	NR	36	61(40-76)	32/4	28/8/0	4.9(0.5-25.8)	13.8(9.3-18.3)	4.3(2.9-13.1)
Lam [30]	Hong Kong	2016	CRC	6.4	45	63(45-80)	32/13	41/4^**^	3.0(1.0-16.0)	7.6(4.2-11.1)	3.9(3.3-4.5)
Schultheis [32]	German	2013	CRC	NR	45	65(18-80)	27/18	27/16/0	3.6(0.1-11.5)	NR	4.0(1.5-11.3)
Sunakawa [36]	Japan	2013	Solid tumor	NR	15	59(34-68)	11/4	12/3/0	2.1(0.9-20.1)	NR	3.7(1.9-12.4)

Abbreviations: CRC, colorectal cancer; GIST, gastrointestinal stromal tumor; HCC, hepatocellular carcinoma; STS, soft tissue sarcoma; PFS, progress-free survival; OS, overall survival. NR, not reported; ECOG PS, European cooperative oncology group performance status; ^*^, non-adipocytic sarcomas;^**^, ECOG 0-1/ECOG 2.

**Table 2:** Number of events reported in every trial included in this study

Author	Year	Underlying malignancy	No. of patients	No. of thrombocytopenia events		No. of anemia events		No. of neutropenia events		No. of Leucopenia events		CTCAE
				All-grade	High-grade	All-grade	High-grade	All-grade	High-grade	All-grade	High-grade	
Li [39]	2015	CRC	136 68	13 1	4 0	5 0	2 0	7 0	3 0	11 0	3 0	4.0
Grothey [38]	2013	CRC	505 255	63 5	14 1	33 6	14 0	NR NR	NR NR	NR NR	NR NR	3.0
Mir [40]	2016	STS	90 92	6 0	1 0	14 9	4 1	3 0	1 0	NR NR	NR NR	4.03
Bruix [37]	2017	HCC	379 194	19 2	8 0	23 2	6 1	NR NR	NR NR	NR NR	NR NR	4.03
Argiles [25]	2015	CRC	53	25	4	12	2	34	21	NR	NR	NR
Masuishi [31]	2017	CRC	146	89	11	109	13	25	4	28	3	4.0
Calcagno [27]	2016	CRC	29	5	2	1	0	3	1	NR	NR	4.01
Del Prete [28]	2017	CRC	136	30	8	NR	NR	NR	NR	NR	NR	4.03
Yeh [34]	2017	GIST	18	18	0	18	2	NR	NR	0	0	4.0
Kim [29]	2015	CRC	32	NR	NR	1	1	1	1	1	1	3.0
Son [33]	2017	GIST	57	16	0	NR	NR	NR	NR	NR	NR	4.0
Zanwar [35]	2016	CRC	23	3	0	6	0	3	0	NR	NR	4.03
Bruix [26]	2013	HCC	36	NR	NR	4	1	NR	NR	NR	NR	3.0
Lam [30]	2016	CRC	45	24	2	21	4	9	0	9	0	4.0
Schultheis [32]	2013	CRC	45	9	2	5	0	22	17	17	4	3.0
Sunakawa [36]	2013	Solid tumor	15	4	0	6	1	NR	NR	4	1	3.0

Abbreviations: CTCAE, common terminology criteria for adverse events; CRC, colorectal cancer; GIST, gastrointestinal stromal tumor; HCC, hepatocellular carcinoma; STS, soft tissue sarcoma; NR, not reported.

### Overall incidence of hematological toxicity

To analyze the overall incidences of hematological toxicities, we considered only arms with regorafenib 160 mg once daily as a single agent and excluded arms with concomitant therapy due to the potential for hematologic toxicities associated with these treatments. Accordingly, a total of 1,569 subjects from 12 non-randomized and single arms of randomized clinical trials were included in this analysis. Two studies [25] [32] were removed because regorafenib was in combination with FOLFOX or FOLFIRI as treatment strategy. The overall incidences of all-grade thrombocytopenia, anemia, neutropenia and leucopenia in subjects receiving regorafenib were 22% (95% CI, 14%-31%), 20% (95% CI, 11%-30%), 10% (95% CI, 4%-14%) and 13% (95% CI, 6%-21%), respectively. The summary incidences of high-grade thrombocytopenia, anemia, neutropenia and leucopenia were 3% (95% CI, 2%-4%), 3% (95% CI, 2%-4%), 2% (95% CI, 1%-3%), 2% (95% CI, 1%-4%), respectively. The test for heterogeneity was significant for all-grade and high-grade thrombocytopenia, anemia, neutropenia and leucopenia (p < 0.1 or I^2^>25%). Therefore, the random-effects model was applied.

### Relative risk of hematologic adverse events

**Figure 2:**
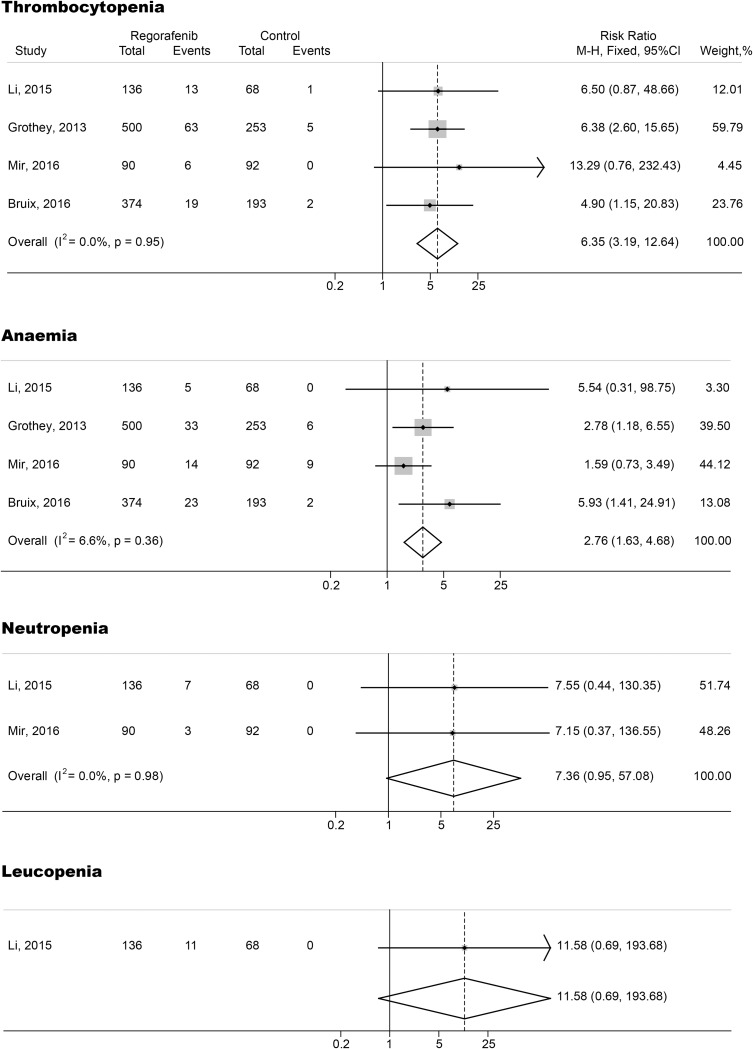
Forest plots of relative risk (RR) of all-grade hematologic toxicities associated with regorafenib versus control The size of squares corresponds to the weight of the trial in the meta-analysis.

**Figure 3:**
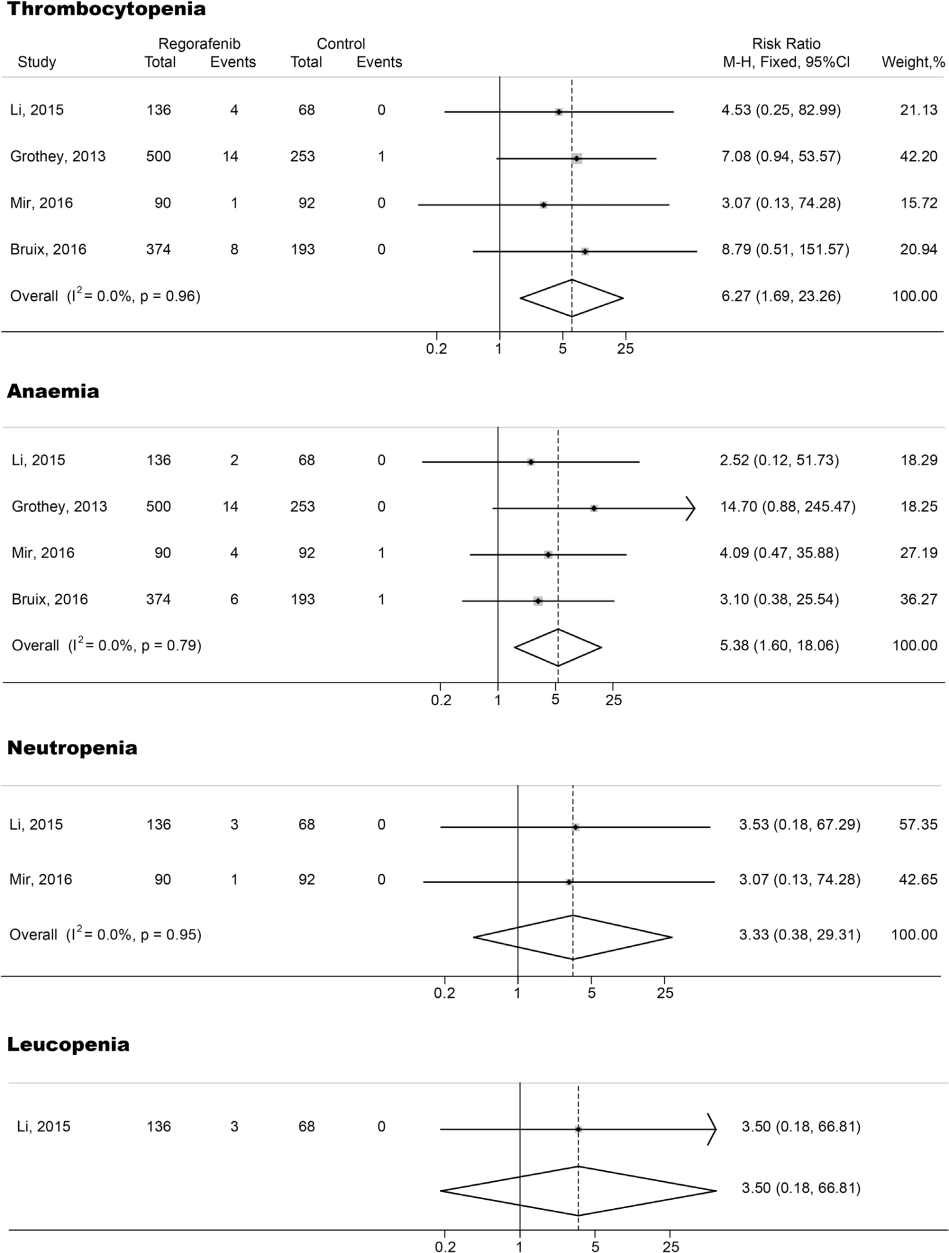
Forest plots of relative risk (RR) of high-grade hematologic toxicities associated with regorafenib versus control The size of squares corresponds to the weight of the trial in the meta-analysis.

The relative risks (RRs) and their 95% CIs of both all-grade and high-grade hematologic toxicities were carried out with four RCTs (three phase III studies and one phase II studies including 1,706 subjects). RRs and their 95% CIs of all-grade thrombocytopenia, anemia, neutropenia and leucopenia were 6.35 (95% CI, 3.19-12.64; p<0.001), 2.76 (95% CI, 1.63-4.68; p<0.001), 7.36 (95% CI, 0.95-57.08; p>0.05) and 11.58 (95% CI, 0.69-193.68; p>0.05), respectively (Figure 2). The relative risks of high-grade thrombocytopenia, anemia, neutropenia and leucopenia in subjects treated with regorafenib were 6.27 (95% CI, 1.69-23.26; p<0.001), 5.38 (95% CI, 1.60-18.06; p<0.001), 3.33 (95% CI, 0.38-29.31; p>0.05) and 3.50 (95% CI, 0.18-66.81; p>0.05), respectively (Figure 3). The fixed-effects model was applied because there was no heterogeneity in the RR analysis of both all-grade and high-grade thrombocytopenia, anemia, neutropenia and leucopenia (p>0.1 and I^2^<25%)

### Publication bias

We found no evidence of publication bias for RRs of both all-grade and high-grade thrombocytopenia, anemia, neutropenia and leucopenia by either Egger or the Begg test (p > 0.05).

## DISCUSSION

To our knowledge, this is the first meta-analysis focusing specifically on hematologic toxicities associated with regorafenib. Our results revealed that the overall incidences of regorafenib-associated all-grade and high-grade (grade 3 and 4) hematologic toxicities, respectively: thrombocytopenia: 22% and 3%; anemia: 20% and 3%; neutropenia: 10% and 2% and leucopenia: 13% and 2%. Furthermore, our analysis from randomized controlled trials demonstrated a significantly increased risk of all-grade and high-grade thrombocytopenia and anemia with regorafenib treatment compared with control. Although not statistically significant, the risks of both all-grade and high-grade neutropenia and leucopenia inclined to increase in regorafenib-treated patients.

Traditional VEGF pathway targeted agents had been linked to a number of mechanism-driven toxicities such as hypertension [6], hepatic toxicity [7], hand-foot skin reaction [8] and arterial thromboembolism [9]. Recently, plenty of data also implied the clinical association of hematological toxicities with these agents such as sunitinib [10], sorafenib [11], bevacizumab [12], although the frequency and severity vary among the different agents (Table 3). It had been demonstrated that a significantly increased risk of both high-grade and all-grade thrombocytopenia and neutropenia was discovered in sunitinib-treated subjects [10] and sorafenib-treated subjects [11]. Bevacizumab, despite not generally treated as a drug prone to cause hematologic toxicities, was also associated with an increased risk of all-grade thrombocytopenia and neutropenia and high-grade neutropenia in a meta-analysis [12]. However, Schutz et al. revealed that sorafenib was associated with a lower risk of high-grade anemia [11] and bevacizumab showed protective effects of both all-grade and high-grade anemia [12]. Our results failed to show a statistically increased risk of all-grade and high-grade neutropenia and leucopenia in regorafenib treated patients. These discrepancies were partly due to the differences in the mechanisms of action among these VEGF-targeted agents, the type of underlying malignancies, insufficient follow-up data and use of blood transfusions among different trials.

**Table 3:** Incidence of hematologic toxicities with anti-angiogenic agents

	All-grade: incidence (95% CI)			High-grade: incidence (95% CI)			References
	Neutropenia	Thrombocytopenia	Anemia	Neutropenia	Thrombocytopenia	Anemia	
Regorafenib	10% (4%-14%)	22% (14%-31%)	20% (11%-30%)	2% (1%-3%)	3% (2%-4%)	3% (2%-4%)	Current study
Sorafenib	18% (15%-22%)	25% (10%-50%)	44% (40%-49%)	5% (3%-8%)	4% (1%-13%)	2% (1%-4%)	[11]
Sunitinib	42% (35%-50%)	45% (37%-53%)	50% (40%-60%)	13% (11%-15%)	11% (9%-13%)	6% (5%-7%)	[10]
Bevacizumab	25% (14%-41%)	14% (8%-23%)	19% (12%-29%)	17% (13%-23%)	3% (2%-6%)	5% (3%-8%)	[12]

It was noted that regorafenib had a biochemical structure similar to sorafenib differing only in the fluorine on the phenyl ring [1, 41]. As showed in Table 3, regorafenib and sorafenib had similar high-grade incidence rates of hematologic toxicities, which were relatively lower compared with other anti-angiogenic agents, such as sunitinib. This was consistent with *in vitro* studies revealing that sunitinib had more activity against both c-KIT and FLT-3 kinases than other inhibitors [42]. However, based on our meta-analysis, regorafenib appeared to have a lower incidence rate of all-grade thrombocytopenia, neutropenia and anemia compared with sorafenib. Although the mechanisms underlying this difference had not been completely explained, it could not rule out that the structural dissimilarity between regorafenib and sorafenib resulted in the different inhibitory effects on angiogenesis related receptors such as VEGFR2 and fibroblast growth factor receptor 1 [1].

The observed hematologic toxicities associated with regorafenib treatment could be explained by the tyrosine kinase inhibition of some hematopoietic growth receptors such as fms like tyrosine kinase 3 (FLT-3) and stem cell factor (c-KIT ligand) [43]. FLT-3 was mainly expressed on committed myeloid, lymphoid precursors as well as the more mature monocytic lineage [44], and its activation played an important role in normal hematopoiesis and cellular growth [45]. FLT and c-KIT ligands, in combination with interleukin-3 (IL-3), had been discovered to regulate the proliferation of hematopoietic progenitor cells [46]. Previous studies had demonstrated that animals with FLT-3 knockout cells displayed a global disruption of hematopoiesis [43]. Our results were consistent with these pre-clinical results and supported the hypothesis that VEGF blockade could increase the risk of myelosuppression.

The hematologic toxicities, especially thrombocytopenia and neutropenia, were one of the most common drug-related adverse events leading to treatment adjustment and discontinuation in clinical trials [28, 30, 31, 34, 38]. High-grade hematologic toxicities were usually clinically significant and required medical intervention. Our study showed that regorafenib-treated subjects had a higher risk of high-grade thrombocytopenia and neutropenia. Although not statistically significant because of limited trials involved in the present study, the relative risk of neutropenia and leucopenia intend to be higher in patients treated with regorafenib. These adverse events could potentially lead to overwhelming sepsis and hemorrhage in patients. In fact, some studies demonstrated fatal adverse events because of bleeding during regorafenib-based therapy [37, 38]. Moreover, it was essential to point out that VEGFR inhibition could cause impairment in the coagulation and endothelial cell function without dysregulating the platelet count [47]. Since we did not have access to any individual patient data, we could not correlate the risks of infection and bleeding with thrombocytopenia and neutropenia. In fact, there were currently no methods to predict subjects at high risk and therefore regular monitoring of complete blood counts was needed. In clinic, because there were no established guidelines in the follow-up and treatment of regorafenib induced high-grade neutropenia and thrombocytopenia yet, temporary dose interruption or dose reduction to 50%-75% of original contents were conducted based on the severity and individual toleration [28, 30, 31, 34].

Here we conducted a comprehensive review using the most up-to-date published data, which made our results more extensive and valid. In addition, with accumulating evidence and enlarged sample size, we had enhanced the statistical power to provide more precise and reliable estimates. However, our study was restricted by some limitations. First, this was a meta-analysis conducted at the trial level and no clinicopathological variables at the patient level could be analyzed. Second, pooled incidence rates had significant heterogeneities. It might be due to the different types of underlying malignancies, sample size, insufficient follow-up data among the included trials. Third, we could not determine the risk of regorafenib-induced hematologic toxicities in different regimens because of the small number of studies available for each regimen. Forth, we could not correlate our data with dose delays and/or interruptions or with hematologic support measures applied. Fifth, different version of Common Terminology Criteria for Adverse Events (CTCAEs) were applied in different trials. However, as far as we know, there were no differences among these versions in term of the definition of hematologic toxicities.

In conclusion, our meta-analysis revealed that regorafenib was associated with an increased risk of hematological toxicities. Clinical doctors should be acknowledged of these potential adverse events and hematologic monitoring at regular intervals might be advised.

## MATERIALS AND METHODS

The present study was conducted in compliance with the recommendations of the *Cochrane Handbook for Systematic Reviews of Interventions* and was reported according to the Preferred Reporting Items for Systematic Reviews and Meta-Analyses (PRISMA) statement [48].

### Literature search and study selection

A comprehensive systematic search of PubMed and Embase database from inception to June 2017 was carried out without any language restrictions. The only keywords used was regorafenib. Both eligibility and exclusion criteria were pre-specified. To be eligible, published trials had to meet the following inclusion criteria: (1) subjects with solid tumor; (2) patients assigned to treatment with regorafenib at a dose of 160 mg orally once daily during weeks 1-3 of each of 4-week cycle; (3) events rates and/or events and sample size available for both all-grade and high-grade (grade 3 and 4) hematologic toxicities including thrombocytopenia, anemia, neutropenia and leucopenia. For incidence study, trials that assigned patients to regorafenib monotherapy were used to define the incidence of hematologic toxicities associated with regorafenib as a single agent. For relative risk study, we included trials that randomly assigned subjects to either placebo or regorafenib in addition to the same treatment to avoid potential confounding factors in the risk of hematologic toxicities. Other publications on the topic, including conference abstract, review articles, pre-clinical papers, editorials, early versions of data later published, articles not dealing with regorafenib were not included (Figure 1). Considering that recent studies with regorafenib might be unpublished, electronic searches were also conducted using the major international congresses’ proceedings (American Society of Clinical Oncology Annual Meeting and European Society of Medical Oncology). Moreover, the reference lists of all studies fulfilling the eligibility criteria were further examined for any relevant studies missed by the electronic searches. When multiple publications of the same clinical trial appeared or if there was a case mix between different publications, only the most recent and/or most complete reporting study was included. Any discrepancies were settled by discussion and consensus.

### Data extraction

Identified abstracts were collected and full texts of potentially relevant studies were reviewed for the trial design and reporting of hematologic toxicities. The following items were extracted from every study: full name of the first author, region, year of publication, underlying malignancy, median follow-up, number of patients for analysis, median age, gender, European cooperative oncology group performance status (ECOG PS), median treatment duration, median overall survival, median progression-free survival (Table 1), number of events of the following adverse events (both all-grade and high-grade): thrombocytopenia, anemia, neutropenia and leucopenia (Table 2). All the reviewers discussed and resolved any discrepancies in the extracted information. The number of subjects evaluated for toxicity was used as the number analyzed for each study, unless it was indicated otherwise. When studies using crossover designs were described, only data available from before the crossover were applied. In cases where this was not available, those trials were not included.

### Statistical analysis

The primary analysis investigated the incidence, relative risk and corresponding 95% CI of all-grade and high-grade hematologic toxicities in cancer patients treated by regorafenib. To calculate the incidence, the number of subjects receiving regorafenib alone and the number of subjects with hematologic toxicities (both all-grade and high-grade) were extracted from the eligible single-arm and randomized controlled trials. The proportion of patients with thrombocytopenia, anemia, neutropenia and leucopenia and 95% CIs were derived from every study. We calculated both RRs and CIs with data extracted only from randomized controlled trials, comparing the incidence of each adverse event in subjects assigned to regorafenib with subjects assigned to control treatment. To calculate 95% CIs, the variance of a log-transformed study specific RR was derived by the delta method. Statistical heterogeneity between different trials and subgroups was assessed by Cochrane’s Q statistic. The I^2^ statistic was calculated to assess the extent of inconsistency contributable to the heterogeneity across different studies [49]. The assumption of homogeneity was considered invalid for I^2^> 25% or p<0.10. Summary RRs and incidences were calculated using fixed-effects or random-effects models depending on the heterogeneity of included trials. Potential publication bias was assessed by visual inspection of a funnel plot, and also evaluated using the tests of Egger et al. [50] and Begg et al. [51]. Two-sided *p* <0.05 were considered statistically significant. All analysis was performed using Stata version 12.0 (StataCorp, USA).
